# Detection of QTLs Regulating Six Agronomic Traits of Rice Based on Chromosome Segment Substitution Lines of Common Wild Rice (*Oryza rufipogon* Griff.) and Mapping of *qPH1.1* and *qLMC6.1*

**DOI:** 10.3390/biom12121850

**Published:** 2022-12-11

**Authors:** Neng Zhao, Ruizhi Yuan, Babar Usman, Jiaming Qin, Jinlian Yang, Liyun Peng, Enerand Mackon, Fang Liu, Baoxiang Qin, Rongbai Li

**Affiliations:** 1 State Key Laboratory for Conservation and Utilization of Subtropical Agro-Bioresources, College of Agriculture, Guangxi University, Nanning 530004, China; 2 Graduate School of Green-Bio Science and Crop Biotech Institute, Kyung Hee University, Yongin 17104, Republic of Korea; 3 Maize Research Institute, Guangxi Academy of Agricultural Science, Nanning 530007, China; 4 State Key Laboratory of Conservation and Utilization of Subtropical Agro-Bioresources, College of Life Science and Technology, Guangxi University, Nanning 530005, China

**Keywords:** agronomic traits, chromosome segment substitution lines (CSSLs), common wild rice (*Oryza rufipogon*), molecular markers, QTL mapping, *qLMC6.1*, *qPH1.1*

## Abstract

Wild rice is a primary source of genes that can be utilized to generate rice cultivars with advantageous traits. Chromosome segment substitution lines (CSSLs) are consisting of a set of consecutive and overlapping donor chromosome segments in a recipient’s genetic background. CSSLs are an ideal genetic population for mapping quantitative traits loci (QTLs). In this study, 59 CSSLs from the common wild rice (*Oryza rufipogon* Griff.) accession DP15 under the indica rice cultivar (*O. sativa* L. ssp. indica) variety 93-11 background were constructed through multiple backcrosses and marker-assisted selection (MAS). Through high-throughput whole genome re-sequencing (WGRS) of parental lines, 12,565 mapped InDels were identified and designed for polymorphic molecular markers. The 59 CSSLs library covered 91.72% of the genome of common wild rice accession DP15. The DP15-CSSLs displayed variation in six economic traits including grain length (GL), grain width (GW), thousand-grain weight (TGW), grain length-width ratio (GLWR), plant height (PH), and leaf margin color (LMC), which were finally attributed to 22 QTLs. A homozygous CSSL line and a purple leave margin CSSL line were selected to construct two secondary genetic populations for the QTLs mapping. Thus, the PH-controlling QTL *qPH1.1* was mapped to a region of 4.31-Mb on chromosome 1, and the LMC-controlling QTL *qLMC6.1* was mapped to a region of 370-kb on chromosome 6. Taken together, these identified novel QTLs/genes from common wild rice can potentially promote theoretical knowledge and genetic applications to rice breeders worldwide.

## 1. Introduction

Rice is a staple food for more than half of the world’s population, and improving its yield is vital for food security. Wild rice (*Oryza rufipogon* Griff.) has always been recognized as the ancestor species of Asian cultivated rice in the evolution, and a natural germplasm resource for generating elite cultivated rice cultivars (*O. sativa* L.) [1,2]. During the process of long-term domestication, many traits of cultivated rice have already been missed by artificial and natural selection. The relatively complete genome of wild rice ensures its wider phenotypic diversity in various traits [3,4].

Grain morphology encompasses grain length (GL), grain width (GW), thousand-grain weight (TGW), which are key factors that affect directly rice yield and quality [5]. The study of wild rice traits, especially grain characteristics, is promising for further improvement of the yield and quality of cultivated rice [6]. To date, more than four genes linked to grain shape have been cloned in rice [7,8,9]. *GW2*, the first major quantitative traits loci (QTL) isolated through map-based cloning, controls the width and weight of rice grains [10]. Similarly, the *GS3* locus controls GL, TGW, GW, and grain plumpness [9], while *GW8/OsSPL16* and *GW7/OsSPL16* are determinants of GW and grain quality [7,8]. Finally, the *GW5* gene has been associated with greater seed vigor and grain yield [11]. The temporary mapping populations such as F_2_ and F_2:3_ populations are sometimes limited by their chaotic genome background and smaller progeny quantity [12], which can only be used for the preliminary characterization and chromosome location of simple phenotypic traits to a large extent [13,14]. With the gradual improvement of plant genetics and genomics research, permanent mapping populations have overcome the shortcomings of temporary populations. Chromosome segment substitution lines (CSSLs) consist of a set of consecutive and overlapping donor chromosome segments in a recipient’s genetic background [15]. They are similar to the near-isogenic lines (NILs) in terms of the ease of identifying new QTLs, since their removal of more genetic interference from the genetic background. Several QTLs have been fine-mapped or cloned based on the NILs in rice [16], and CSSLs have been recently used to clone the GL-controlling gene *GL4* [17]. TGW is one of the major factors determining rice yield, but the molecular and genetic factors that determine rice grain weight is still unclear [18]. Identification and utilization of major QTLs associated with rice TGW are of great significance [19]. In recent years, breakthroughs in the advanced genome-wide association analysis (GWAS) methods promote the identification of several new candidate genes and alleles linked to TGW in rice [20]. The *GSK2-LARGE1/OML4* pathway controlling TGW in rice has been discovered, which revealed that the TGW is determined by the interaction of many alleles and QTLs [21,22]. The GLWR is one of the main determinants of the rice grain shape. At present, several GLWR-related genes have been cloned from rice. For instance, the *OsRA2* RNA interference plant showed an increased grain length-width ratio (GLWR) [23]. Compared with wild-type rice, the GLWR and TGW of *OsGRF1* overexpression plants were higher than *Osgrf1* [24]. Furthermore, some grain quality-related genes have been cloned from common wild rice. For instance, *Bh4* was identified in the common wild rice line W1943 and controlled the black glume color [25]. *Gh1* encodes chalcone isomerase, and its mutation results in the golden glume phenotype [26]. In addition, the reddish glume and internode gene *Gh2*, which encodes a cinnamyl alcohol dehydrogenase (CAD), is a key enzyme in plant secondary metabolism and lignin synthesis [27]. *Pb* (also known as *Prp-b*) and its complementary gene *Pp* (also known as *Prp-a*) are responsible for the purple seed coat [28]. *Ra* and *Rb* genes with basic helix-loop-helix (bHLH) structure and homology to *Lc* were isolated from the leaf cDNA library of a purple leaf rice variety. The leaf margin color (LMC) is a distinctive trait in wild rice compared to cultivated rice, and a potential phenotypic trait that can be used as a marker by rice breeders to screen hybrids [29,30].

The economic traits identified from wild rice such as the plant height (PH) are important plant architecture traits to improve rice yield and biomass [31]. Semi-dwarf (*sd-1*), a defective gibberellin 20-oxidase gene in the gibberellin (GA) synthesis pathway, generated semi-dwarf rice and set a new record of rice yield in Asia in the 1960s [32]. As a major semi-dwarf gene, *sd-1* is still widely used in modern rice varieties [33]. *SLR1* gene encodes a DELLA protein, which is a negative regulator in the process of gibberellin GA signal transduction. Its *slr1* mutant shows weak stems and elongated basal internodes [34]. As a BR signal receptor, *OsBRI1* is highly homologous with *AtBRI1* gene in Arabidopsis. It regulates rice internode elongation mainly by controlling cell division and elongation [35]. Internode elongation in rice is mainly controlled by phytochromes, which can regulate the expression of the GA biosynthetic gene *OsGA3ox2* affecting the elongation of rice internodes [36,37]. Rice cultivars of higher PH are economic and environmental-friendly in maintaining both plant production and animal husbandry in paddy fields [38,39,40]. Thus, many PH-related QTLs of rice have been already detected by researchers worldwide [41,42].

Aiming at improving the efficiency of novel QTLs detection and promoting the rice breeding practice, the DP15-CSSLs were constructed by multiple backcrossing, self-crossing, and marker-assisted selection (MAS) from the wild rice DP15 and 93-11 in this study [43]. The 255 pairs of molecular markers developed evenly distributed across 12 chromosomes were to establish a set of CSSLs of wild rice covering the whole DP15 genome. In addition, 20 grain-related QTLs, one PH-related QTL *qPH1.1,* and one LMC-related QTL *qLMC6.1* were detected according to the DP15-CSSLs, which were promising for the identification of new QTLs/Genes. The dominant QTL locus *qPH1.1* controlling higher PH was mapped and characterized based on DP15-CSSLs and will be meaningful to explain the formation mechanism of higher PH in wild rice. The *qLMC6.1* associated with purple leaf margin of wild rice was located in a region of 370-kb on chromosome 6 by the DP15-CSSLs, and *qLMC6.1* can also control the leaf sheath color (LSC), stigma color (SC), and apiculus color (AC) was characterized to explore the distribution of anthocyanin in putative tissues and cells. Taken together, the DP15-CSSLs are a repository of various traits of Guangxi common wild rice, which can be effectively used as the introgression lines of wild rice in generating improved hybrid rice cultivars and ideal genetic populations for QTLs/genes mapping [44].

## 2. Materials and Methods

### 2.1. Plant Materials

**Figure 1:**
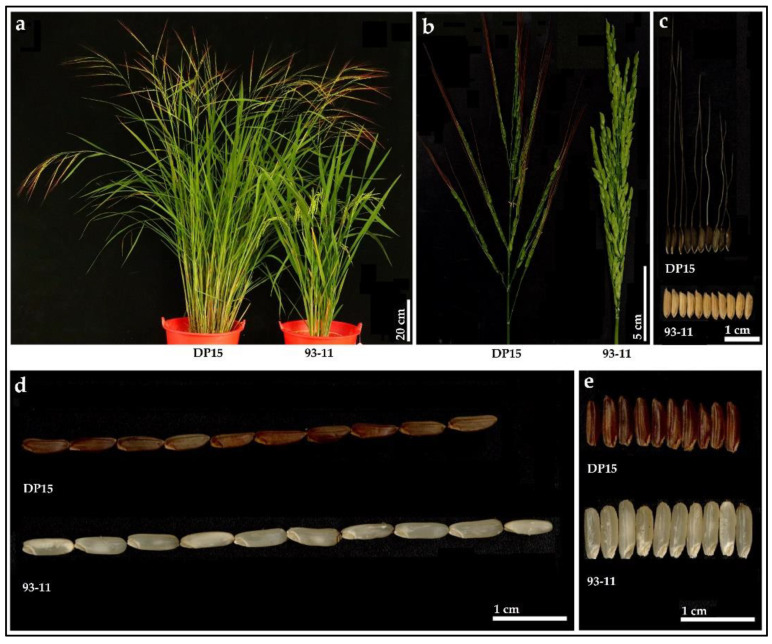
Phenotypic morphology of DP15 and 93-11. (**a**), plant phenotype of DP15 and 93-11, bar = 20 cm; (**b**), panicle morphology of DP15 and 93-11, Bar = 5 cm; (**c**), phenotype of mature grain of DP15 and 93-11, bar = 1 cm; (**d**), length of brown rice of DP15 and 93-11, bar = 1 cm; (**e**), width of brown rice of DP15 and 93-11, bar = 1 cm.

In the present study, one elite wild rice accession DP15 was screened to establish wild rice CSSLs from 2361 Guangxi common wild rice materials preserved in the nursery of State Key Laboratory for Conservation and Utilization of Subtropical Agro-Bioresources, Nanning, Guangxi Province, China (Figure 1 and Figure S1) [15]. As a representative Guangxi common wild rice accession, DP15 shows various plant traits that are significantly different from cultivated rice variety 93-11 (Figure 1 and Figure S1, and Table S1). DP15 was used as a donor parent and 93-11 receptor parent to develop DP15-CSSLs (Figure 1 and Figure S2, Tables S2 and S3). The DP15-CSSLs materials and its parental materials used for QTLs detection and mapping were grown during 2020 (from 20 November 2019 to 10 May 2020) in Sanya, China (109°14′12″ N, 18°08′49″ E), and 2021 (from 2 March 2021 to 10 July 2021) in Nanning, Guangxi Province, China (109°01′16″ N, 22°79′98″ E), respectively.

### 2.2. Development of the DP15-CSSLs

**Figure 2:**
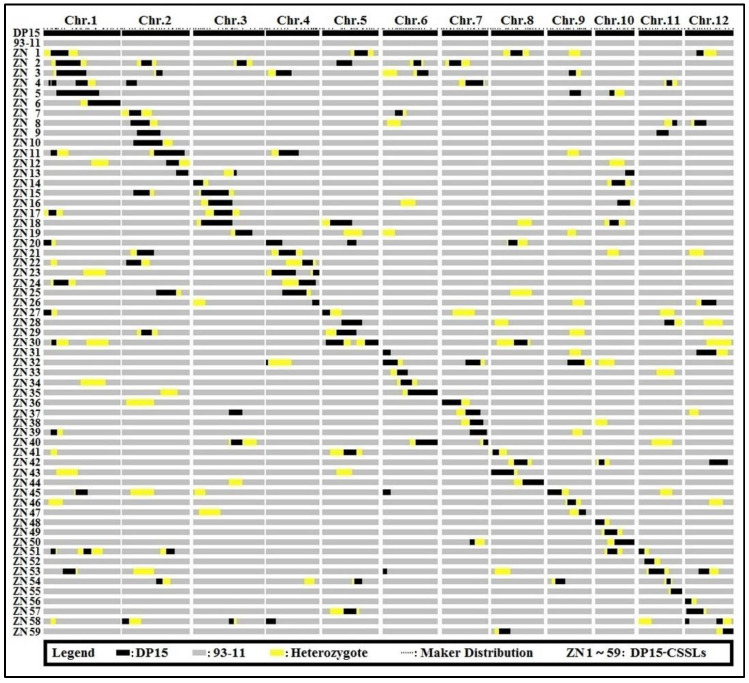
Graphical genotypes of the 59 lines DP15-CSSLs. Note: Black bars indicate homozygous chromosome substituted segments derived from DP15; Yellow bars indicate heterozygous substituted segments derived from DP15; Grey bars indicate the genetic background of recipient parent 93-11.

The DP15 and 93-11 were respectively used as the donor and recipient parents to develop a set of CSSLs [15]. The DP15-CSSSLs were constructed by MAS, backcrossing, phenotypic identification, and artificial selections, as described previously (Figure 1 and Figure S2, Tables S2 and S3) [43,44,45,46]. The F_1_ progeny derived from a cross between 93-11 and DP15 were then backcrossed with 93-11 to produce the BC_1_F_1_. The self-crossed progeny of BC_2_F_1_ were selected based on 255 polymorphic molecular markers evenly distributed across 12 chromosomes (Figure 1 and Figure S2, Tables S2 and S3). BC_2_F_1_ plants were then backcrossed with 93-11 to produce the BC_3_F_1_ progeny, and primary CSSLs were selected based on their genotypes by MAS, which were then self-crossed to obtain the BC_4_F_1_ generation. Candidate CSSLs were screened from these progenies to detect the residual donor segments, followed by the selection of BC_5_F_1_ lines. The latter were then backcrossed to generate the BC_6_F_1_ progeny. Finally, the overlapping substitution segments across different lines were also screened from the BC_4_F_2_, BC_5_F_2_, and BC_6_F_2_ progeny to obtain the definite CSSLs (Figure 2 and Figure S2, and Table S4).

### 2.3. The Methods of Phenotypic Characterization of DP15-CSSLs

**Figure 3:**
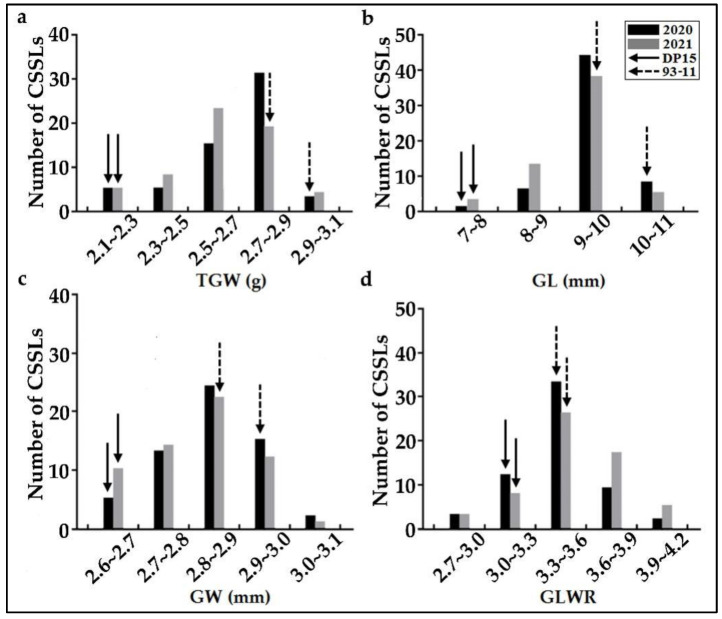
Frequency distribution of 59 DP15-CSSLs and their two parents on four grain traits over two years. Note: (**a**), phenotypic distribution of TGW in DP15, 93-11, and the DP15-CSSLs; (**b**), phenotypic distribution of GL in DP15, 93-11, and the DP15-CSSLs; (**c**), phenotypic distributions of GW in DP15, 93-11, and the DP15-CSSLs; (**d**), phenotypic distribution of GLWR in DP15, 93-11, and the DP15-CSSLs; The marked solid arrow lines and dotted arrow lines indicate the phenotypic distribution of DP15 and 93-11 respectively.

The DP15-CSSLs and its parent lines were planted with a 20-cm plant-spacing and a 30 cm row-spacing in the experimental field. Three randomly selected biological replicates of each DP15-CSSLs line were statistically measured for the values of various traits. The phenotypic characteristics of the DP15-CSSLs mature plants, including grain number (GN), TGW, GL, GW, GLWR, PH, LMC, AC, and LSC, were recorded under natural conditions in the experimental field during 2020 (from 20 November 2019 to 10 May 2020) in Sanya, China, and 2021 (from 2 March 2021 to 10 July 2021) in Nanning, Guangxi Province, China, respectively (Figure 3, Figures S2 and S3, and Table S5). When the seeds were matured under natural conditions, the dry seeds of each line were selected to measure the values of grain traits as previously described [47]. The tissue slices were processed by the frozen section machine Hestion CM2850. The vascular bundle scanning pictures were captured by a scanning electron microscope SEM refers to previous research [48]. The cell pictures were obtained by paraffin section with a phloroglucinol staining according to established protocols [49]. The anthocyanin fluorescence images of rice stigma protoplasts were captured by the confocal microscope (Leica-TCS-SP8MP) in reference to previous experimental methods [50].

### 2.4. Whole Genome Re-Sequencing (WGRS) and Bioinformatic Analysis of Genomic SSR, InDel, and SNP Markers

The genomic DNA of DP15 and 93-11 were extracted using a kit (Rapid Plant Genomic DNA Isolation Kit, Sangon Biotech). The SSR markers used in this research were referenced to the SSR database of rice (https://archive.gramene.org/db/markers; accessed on 15 November 2016) (Tables S2 and S3). The whole genome re-sequencing (WGRS) was performed on an Illumina HiSeq2500™ by Novogene Company (Beijing, China) refers to the standard Illumina protocol (Figures S5 and S6, and Table S6) [51]. The FASTQ files were processed by the software of FASTQ version 0.6.0 to access the read quality (https://github.com/OpenGene/fastp; accessed on 3 June 2019). The WGRS data were then compared to design the whole genome SNP (Single nucleotide polymorphism) markers and InDel (Insertion-deletion) markers according to the website of BWA version 0.7.16 (http://bio-bwa.sourceforge.net/; accessed on 4 August 2019) (Figures S7 and S8). Polymorphic genomic sites were evenly selected and designed for InDel markers (Figures S7 and S8, and Table S7). The Polymorphic regions (≥1 bp variation) with high sequencing depth (DP15 ≥ 50 fold) were selected to design the SNP and InDel markers [52]. The circos software online was also used to visualize the SNPs and InDels (http://www.circos.ca/software/download/circos/; accessed on 6 August 2019). The primers were designed by the online software Primer3 version 0.4.0 tools (https://bioinfo.ut.ee/primer3-0.4.0/; accessed on 7 August 2019). The PCR product size was designed with a range from 200 to 500 bp (Figure S8, Tables S1–S3 and S7).

### 2.5. Genomic DNA Extraction and PCR Amplification

Genomic DNA of DP15-CSSLs was extracted using a modified version of the CTAB method [53], and amplified by PCR according to established protocols [54]. The PCR products were separated in 7% polyacrylamide denaturing gels, and the bands were visualized using the silver-staining method and genotyped as previously described (Figure S9) [55].

### 2.6. QTL Mapping and Data Analysis

The substituted segments in DP15-CSSLs were screened as described previously [15]. The DP15-CSSLs genomes were visualized graphically using the Graphical Geno-Types32 software (GGT32), and putative QTLs were identified based on the significance level of *p* ≤ 0.001. If several CSSLs with overlapping substituted segments exhibit similar phenotypes, the relevant QTL is likely localized to an inter-genomic interval (Figure 2 and Figure S10, and Table S4) [56]. Based on the SNP markers located in target regions of QTLs, a BSA (Bulk segregation analysis) method based on GSR40K gene chip technology is used for fine mapping and verification of target QTLs [57]. The QTL nomenclature was followed as the previously described method [58], and the linkage map of QTLs was constructed using the Map-Chart 2.2 software [59]. The phenotypes and genotypes of the CSSLs were finally evaluated by the QTL Ici-Mapping 4.1.0 software, and the QTLs were mapped by a permutation test (Permutations = 1000, *p* = 0.05) [60]. The additive effect of a QTL was calculated as (Phenotypic value of CSSLs—phenotypic value of 93-11)/2, and the phenotypic contribution ratio of the additive effect was calculated as (Additive effect value/phenotypic value of 93-11) × 100 (Figure 2, Figure 3, Figures S10 and S11, and Table S4).

## 3. Results

### 3.1. Whole Genome Re-Sequencing (WGRS) of the Parental Materials

The parental material DP15 and 93-11 were re-sequenced by Illumina high-throughput sequencing technology by Novogene Company (Beijing, China), and the resultant genome sequence data were then mapped by the IRGSP-1.0 software. These high-quality sequencing data of 9.68 G and 9.8 G were obtained from DP15 and 93-11 genome re-sequencing with average sequencing depths of 19.37× and 20.45×, respectively (Table S6). The numbers of mapped reads between DP15 and 93-11 genome were 52,922,056 and 50,987,541, respectively (Figures S5 and S6). The GC content of mapped reads between DP15 and 93-11 was 45.3% and 44.2% (Table S6). The SNPs between DP15 and 93-11 genome are mainly mapped to the coding regions (CDS) and the 5′, 3′ untranslated regions (5′, 3′ UTR) with a total percentage of 95.11% to the whole genome variation (Figures S5 and S6). Compared to the Nipponbare reference genome, the numbers of SNPs in the DP15 and 93-11 genomes were 1,894,103 and 690,409, respectively (Figure S7 and Table S6).

### 3.2. Selection of Polymorphic Markers between DP15 and 93-11 Genome

The average genome SNPs density of DP15 and 93-11 were 0.51634603% and 0.182119873%, respectively. The frequency distribution and density of polymorphic genome SNPs and InDels between DP15 and 93-11 were detected, respectively. Based on the comparative analysis of the whole genome re-sequencing data of the parent materials DP15 and 93-11, 15,691 polymorphic InDel loci were detected between the two parental genomes. Among them, 12,565 mapped InDels were designed for polymorphic molecular markers by online bioinformatic software Primer3-0.4.0 (https://bioinfo.ut.ee/primer3-0.4.0/; accessed on 7 August 2019). The melting temperatures of the InDels were 55.02~61.24 °C, the GC content was 26.9~72.2%, and the average product lengths of the InDel markers were from 100 bp to 500 bp (Figures S7 and S8, and Table S1). Combining the InDel primers with the 2261 pairs of rice genomic SSR primers available in the laboratory, a total of 255 pairs of polymorphic markers with an average distance of 1.47 Mb were developed (Figure S8; Tables S1, S6, and S7). The representative electropherogram showed that there are polymorphic bands amplified with these developed InDels and SSR markers (Figure S9).

### 3.3. Chromosome Substitution Segments Analysis of DP15-CSSLs

In total, 255 pairs of genomic molecular markers have been developed with an average distance of about 1.47 Mb between two adjacent markers to establish these DP15-CSSLs (Tables S2 and S3). In this study, 59 CSSLs harboring targeted DP15 chromosomal segments in the 93-11 genetic background were finally established. The estimated length of the substituted chromosome segments in DP15-CSSLs ranged from 1.1 Mb to 15.9 Mb with an average length of 7.5 Mb. The cumulative coverage length of DP15-CSSLs segments is 344.34 Mb. Most of the complete genomes in each chromosome were covered by the DP15-CSSLs except for chromosomes 1 (80.37%), 2 (92.89%), 3 (82.14%), 4 (89.63%), 5 (92%), 7 (88.56%), 9 (92.75%), and 12 (97.14%). The total coverage rate of substituted segments in a genome was 91.72%, the average coverage rate of substitution segments in a chromosome was 92.96%, and the highest and lowest coverage was seen with chromosomes 6 (100%) and 1 (80.37%), respectively (Figure 2 and Figure S10, Tables S3 and S4).

### 3.4. Characteristics of Four Grain Related Traits of the DP15-CSSLs

**Figure 4:**
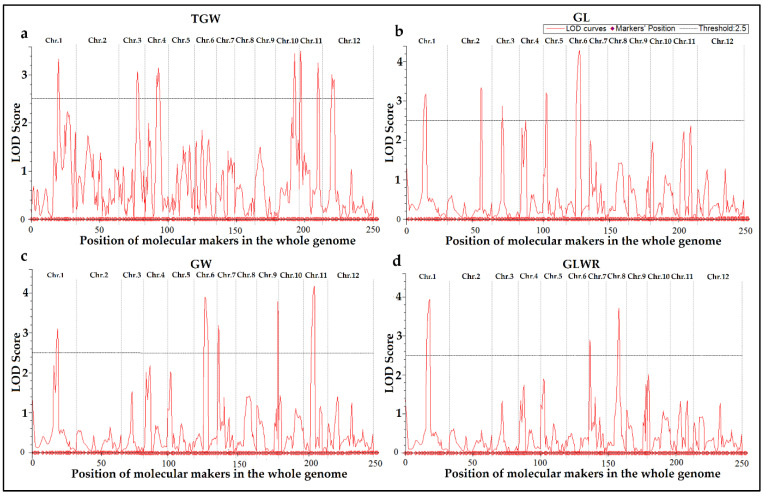
Detection of QTLs related to four grain traits in the DP15-CSSLs. (**a**), detection of QTLs related to TGW in DP15-CSSLs; (**b**), detection of QTLs related to GL in DP15-CSSLs; (**c**), detection of QTLs related to GW in DP15-CSSLs; (**d**), detection of QTLs related to GLWR in DP15-CSSLs.

The phenotypic variations between the parent lines and DP15-CSSLs were recorded, respectively, in Sanya and Naning during 2020 and 2021. The phenotypic values on four grain traits GL, GW, TGW, and GLWR were statistically analyzed, and the results showed that the phenotypic values collected from the two experimental sites over two years were consistent except for slight variation. The phenotypic values of GL, GW, TGW, and GLWR in DP15-CSSLs and its parents showed an extensive variation, which implied that there might be potential QTLs to be identified (Figure 3 and Figure S11, Tables S5 and S8). Compared to their two parents, these DP15-CSSLs showed a higher GLWR over the two years, which will also lay a foundation for the mapping of novel QTLs and the breeding of new cultivars. In addition, DP15-CSSLs showed a higher GL and GW in Sanya during 2020 than in Nanning during 2021, which may be affected by the environmental and ecological conditions. Based on the phenotypic values recorded, QTLs analysis on DP15-CSSLs ware carried out for the four grain traits TGW, GL, GW, and GLWR, about 20 QTLs were finally detected with linked molecular markers in the DP15-CSSLs (Figure 3, Figure 4, Figures S10, and S11, Tables S5 and S8).

### 3.5. Identification and Detection of TGW-Related QTLs of DP15-CSSLs

Combing the genotypes and TGW phenotype of DP15-CSSLs, QTLs related to TGW were screened in this research (Figure 3a, Figure 4a, and Figure S11, Tables S5 and S8). Most of the TGW in DP15-CSSLs were significantly less than that of 93-11 over the two years of 2020 and 2021 (Table S5). Seven QTLs (*qTGW1.1*, *qTGW3.1*, *qTGW4.1*, *qTGW10.1*, *qTGW11.1*, *qTGW11.2*, and *qTGW12.1*) related to TGW were identified with linked molecular markers on Chromosome 1, 3, 4, 10, 11, and 12, respectively (Figure 4a and Figure S11, and Table S8). Furthermore, *qTGW1.1* showed a linkage with a molecular marker DXB-1-3 in chromosome one. The *qTGW3.1* was identified in the overlapped substitution segments of ZN15, and ZN16 between the SSR marker RM517 and RM251, where the previously cloned gene *LPA1* is also located [61]. The *qTGW4.1* was mapped between RM1018 and RM3288. Likewise, *qTGW10.1* was identified in the overlapping segment DP15-CSSLs of ZN49, and ZN50 within the RM6404 to RM216 region (Figure 4a and Figure S11, and Table S8). There are two QTLs related to TGW were detected in chromosome 11, named *qTGW11.1 and qTGW11.2.* The *qTGW12.1* detected in chromosome 12 was located in an overlapping segment of ZN57 and ZN58 near the simple sequence repeat (SSR) marker RM313. The phenotypic contribution ratios of the seven TGW-related QTLs ranged from 7.96~11.11% in 2020 and 9.24~12.61% in 2021 (Figure 3a, Figure 4a, and Figure S11, and Table S8).

### 3.6. Identification and Detection of GL-Related QTLs of DP15-CSSLs

Some of the DP15-CSSLs showed shorter GL than 93-11 over two years (Figure 3b and Table S5). Five effective QTLs (*qGL1.1*, *qGL2.1*, *qGL3.1*, *qGL5.1,* and *qGL6.1*) related to GL were respectively identified on Chromosome 1, 2, 3, 5, and 6 (Figure 4b and Figure S11, Table S8). The *qGL1.1* were linked to the over lapping segment of ZN4 and ZN5. The *qGL2.1* was located at an interval between RM6842 and RM12355. The *qGL3.1* in the DP15-CSSLs of ZN18 is linked with the marker RM1164. The *qGL5.1* was identified as co-segregated with the RM1200 in the overlapping segment of ZN18 and ZN27. Finally, *qGL6.1* was identified in the ZN35 substituted region between RM6071 and RM400 (Figure S11 and Table S8). The CP values of *qGL3.1*, *qGL5.1,* and *qGL6.1* ranged from 11.52% to 6.5% in 2020 and from 11.94% to 6.5% in 2021 (Figure 4b and Figure S11, Table S8).

### 3.7. Identification and Detection of GW-Related QTLs of DP15-CSSLs

The GW of five DP15-CSSLs (ZN6, ZN35, ZN39, ZN40, ZN46, and ZN51) were significantly different from that of 93-11(Figure 3c, and Tables S5 and S9). Five QTLs (*qGW1.1*, *qGW6.1*, *qGW7.1*, *qGW9.1,* and *qGW11.1*) related to GW were identified that were distributed on Chromosome 1, 6, 7, 9, and 11, respectively. While *qGW1.1* was identified between RM10159 and RM486, *qGW6.1* was located between RM6071 to RM400, where the previously cloned gene *DSG1* is also located [62]. In addition, *qGW7.1* was in the substituted segments of ZN39 and near RM429, *qGW9.1* was mapped to a substituted region of ZN46 between RM257 and RM245, and *qGW11.1* in ZN51 between RM167 and RM120 (Figure 4c and Figure S11, and Table S8). The CP values ranged from 7.96% to 6.89% in 2020 and from 6.35% to 5.37% in 2021 (Table S8).

### 3.8. Identification and Detection of GLWR-Related QTLs of DP15-CSSLs

The GLWR of three CSSLs (ZN6, ZN40, and ZN43) was larger than that of 93-11 in 2020 (Figure 3d, Tables S5 and S8). Three QTLs (*qGLWR1.1*, *qGlWR7.1*, and *qGLWR8.1*) related to GLWR were identified, of which *qGLWR1.1* was located in the segment of ZN6 between RM297 and RM486. The average CP value of *qGLWR1.1* was 7.9% in 2020 and 8.15% in 2021 (Table S8). In addition, *qGlWR7.1* was located near RM429, and its CP values were 8.51% in 2020 and 8.77% in 2021 (Figure 4d and Figure S11, and Table S8). Finally, *qGLWR8.1* was located in the overlapping segment of ZN43 and ZN53 between RM344 and RM5663, and its mean CP values in 2020 and 2021 were 11.55% and 11.69%, respectively (Table S8).

### 3.9. Identification and Genetic Mapping of the qPH1.1

#### 3.9.1. Characterization of the PH of a DP15-CSSL Line

**Figure 5:**
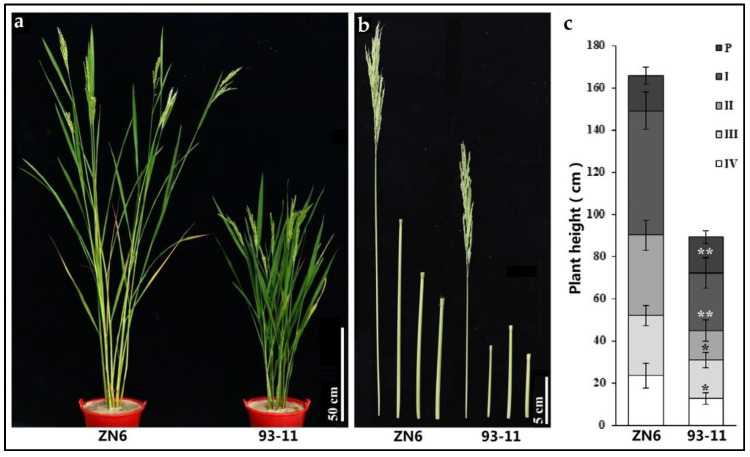
Phenotype of a PH-related DP15-CSSL line. (**a**), the plant architecture of ZN6 and 93-11 at heading stage, bar = 50 cm; (**b**), the internode of ZN6 and 93-11 at heading stage, bar = 5 cm; (**c**), the histogram of internode length of ZN6 and 93-11 at heading stage; the sign “*” in Figure 5c indicates a *p* ≤ 0.05 level; The sign “**” in Figure 5c indicates a *p* ≤ 0.01 level.

ZN6 is a homozygous DP15-CSSL line, the chromosomal substitution segments of ZN6 were located on chromosome 1. The internode length of ZN6 and 93-11 were statistically counted at the maturation stage. The typical phenotype of ZN6 is a higher stem, and its PH is significantly higher than that of its recipient parent 93-11. Through the comparison of phenotypic values between ZN6 and 93-11 on the internode traits, the results showed that both the length and diameter of the first, second, third, and fourth internode of the substitution line ZN6 significantly increased compared with the donor parent 93-11 except for the panicle length (Figure 5 and Table S9). It can be inferred that the QTL controlling the longer stem of wild rice is located between two pairs of primers RM5 and DXB-1-7 on rice chromosome 1 (Figure 1 and Figure 5, and Table S9).

#### 3.9.2. Characterization of the Cell Morphology in Culm

**Figure 6:**
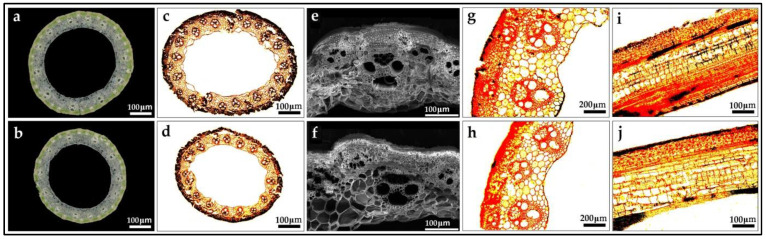
Tissue section, vessel scanning, and paraffin section figures of the uppermost internode at the grain filling stage. (**a**), the tissue section figure of the uppermost internode of ZN6, bar = 100 μm; (**b**), the tissue section figure of the uppermost internode of 93-11, bar = 100 μm; (**c**), the horizontal paraffin section figure of the uppermost internode of ZN6, bar = 100 μm; (**d**), the horizontal paraffin section figure of the uppermost internode of 93-11, bar = 100 μm; (**e**), the horizontal SEM section figure of the uppermost internode of ZN6, bar = 100 μm; (**f**), the horizontal SEM section figure of the uppermost internode of 93-11, bar = 100 μm; (**g**), the horizontal paraffin section figure of the uppermost internode of ZN6 and 93-11 at heading stage, bar = 200 μm; (**h**), the horizontal paraffin section figure of the uppermost internode of ZN6 and 93-11 at heading stage, bar = 200 μm; (**i**), the longitudinal paraffin section figure of the uppermost internode of ZN6, bar = 100 μm (**j**), the longitudinal paraffin section figure of the uppermost internode of 93-11, bar = 100 μm.

The length of rice stem is mainly determined by two main factors: cell length and cell numbers in unit area [63]. Therefore, the internodes of ZN6 and 9311 at the grain-filling stage were selected for tissue section analysis respectively. The results of the cell section showed that there was no significant difference in cell size between ZN6 and the recipient parent 93-11, but the cell density per unit area of ZN6 was significantly larger than that of the donor parent 93-11. This result preliminarily shows that the cell density per unit area of ZN6 was increased by genes that regulate the course of cell division, which finally promotes a higher PH and a thickening stem phenotype (Figure 6 and Table S10).

#### 3.9.3. Genetic Analysis and Mapping of the *qPH1.1*

**Figure 7:**
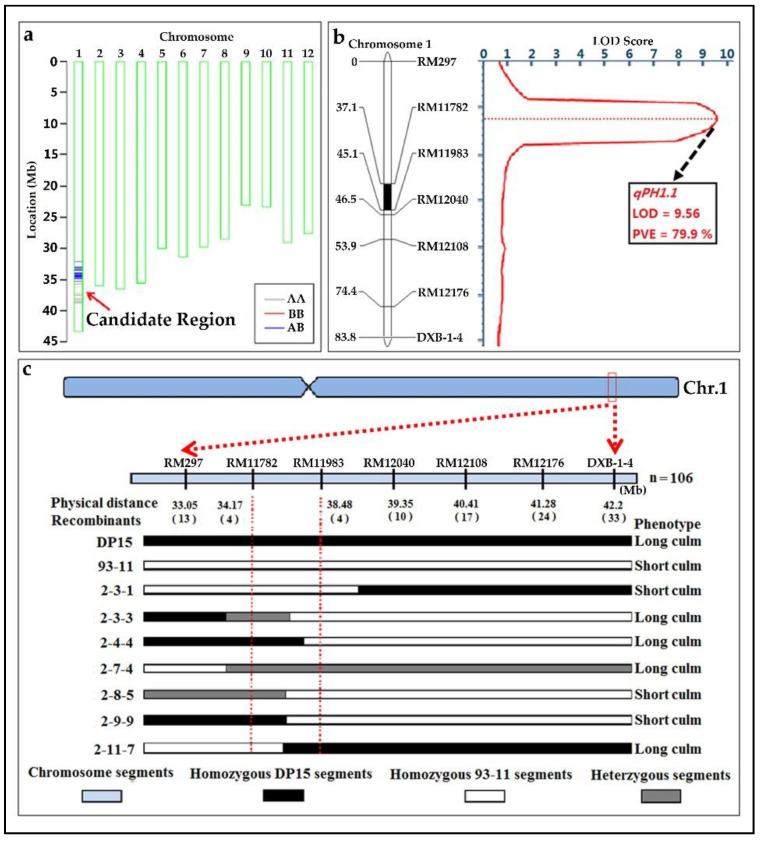
Genetic mapping of the *qPH1.1* in rice. (**a**), the QTL detection figure of *qPH1.1*; (**b**), the BSA analysis for *qPH1.1*; (**c**), the recombinant identification and genetic mapping for *qPH1.1*.

Through the genome background analysis of the ZN6, it can be inferred that the QTL controlling the higher stem of wild rice is located between two pairs of primers RM5~DXB-1-4 on rice chromosome 1 (Figure 7 and Table S2). For precise identification and mapping of the *qPH1.1* controlling long culm in ZN6, a secondary genetic population was constructed by the backcross between ZN6 and recipient parent 93-11. The phenotype and genotype of each individual in the secondary F_1_ and F_2_ population were statistically recorded for the genetic mapping and fine mapping for the *qPH1.1*. The results showed that all the individuals in the F_1_ generation exhibited a higher PH phenotype, and the PH phenotype in the F_2_ population was obviously separated, then the phenotype data of the F_2_ population were calculated for genetic analysis (Table S11). Almost 82 of the 106 F_2_ plants exhibited long culm phenotype, and 24 plants showed short culm phenotype, which was consistent with the Mendelian 3:1 segregation ratio (χ^2^ = 0.101 ≤ χ^2^ _0.05,1_ = 3.84) (Table S11). Thus, *qPH1.1* is likely encoded by a single dominant QTL. *qPH1.1* was further located in an overlapping segment between SNP marker R0130491732 (30.49 Mb) and F0138403159 (40.41 Mb) by a BSA method with 40K SNP microarrays Chips (Figure 7a and Table S2). Based on the results of high-throughput sequencing, seven pairs of polymorphic molecular markers identified from the 12,565 mapped InDels were selected to do a fine mapping (Tables S1, S2, and S12). The genotypes and phenotypes of the F_2_ population were identified, which confirmed that *qPH1.1* was located in the 4.3-Mb region between RM11782 and RM11983, with a LOD value of 9.56, a PVE value of 79.9% (Figure 7b,c, Tables S1 and S12).

### 3.10. Identification and Genetic Mapping of the qLMC6.1

#### 3.10.1. Characterization of the LMC of a DP15 CSSLs Line

**Figure 8:**
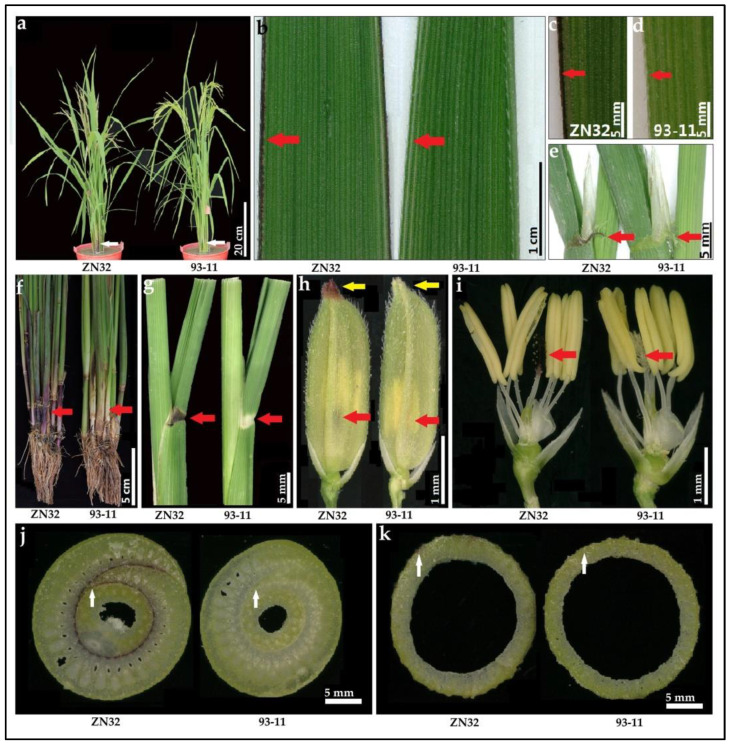
Phenotype of ZN32, an LMC-related DP15-CSSL line. (**a**), the plant architecture of ZN32 and 93-11 at heading stage, bar = 20 cm; (**b**–**d**) in Figure 8 show the leaf margin morphology of ZN32 and 93-11 at heading stage, the red arrow in (**b**–**d**) indicates the leaf margin site, the bar in (**b**–**d**) are 1 cm, 5 mm, and 5 mm, respectively; (**e**), the ligule and auricle color of ZN32 and 93-11 at heading stage, the red arrow in (**e**) shows the auricle site, the scale bar = 5 mm; (**f**), the basal shoot of ZN32 and 93-11 at heading stage, the red arrow in (**f**) shows the basal shoot region, bar = 5 cm; (**g**), the leaf collar phenotype of ZN32 and 93-11 at heading stage, the red arrow in (**g**) shows the lamina joint site, bar = 5 mm; (**h**), the apiculus color of ZN32 and 93-11 at heading stage, the yellow arrow in (**h**) shows apiculus site; the red arrow shows stigma site, bar = 1 mm; (**i**), the stigma color of ZN32 and 93-11 at heading stage; The red arrow shows stigma site, bar = 1 mm; (**j**), the rice basal culm with leaf sheath surrounded of ZN32 and 93-11 at heading stage, the white arrow in (**j**) shows the zone of inner leaf sheath, bar = 5 mm; (**k**), the rice basal culm of ZN32 and 93-11 at heading stage, the white arrow in Figure 8k shows the borders of the culm, bar = 5 mm.

ZN32 is a homozygous DP15-CSSL line that contains DP15 substitution fragments in chromosome 6. ZN32 shows a purple leaf margin phenotype that is significantly different from 93-11 (Figure 8 and Figure S11). Besides the LMC, the phenotypes related to plant architecture, leaf sheath, culm, auricle, apiculus, stigma, and basal shoot were identified. The results showed that there are significant differences between ZN32 and the recipient parent 93-11 among the color of the leaf margin, basal shoot, pillar, auricle, apiculus, stigma, and so on (Figure 8 and Figure S11). These results show that the differences between ZN32 and 93-11 are significant and stable, which implies that there is a gene controlling LMC located in chromosome 6 (Figure 2 and Figure S11, and Table S4).

#### 3.10.2. Characterization of the Cell Morphology in Stigma Cell

**Figure 9:**
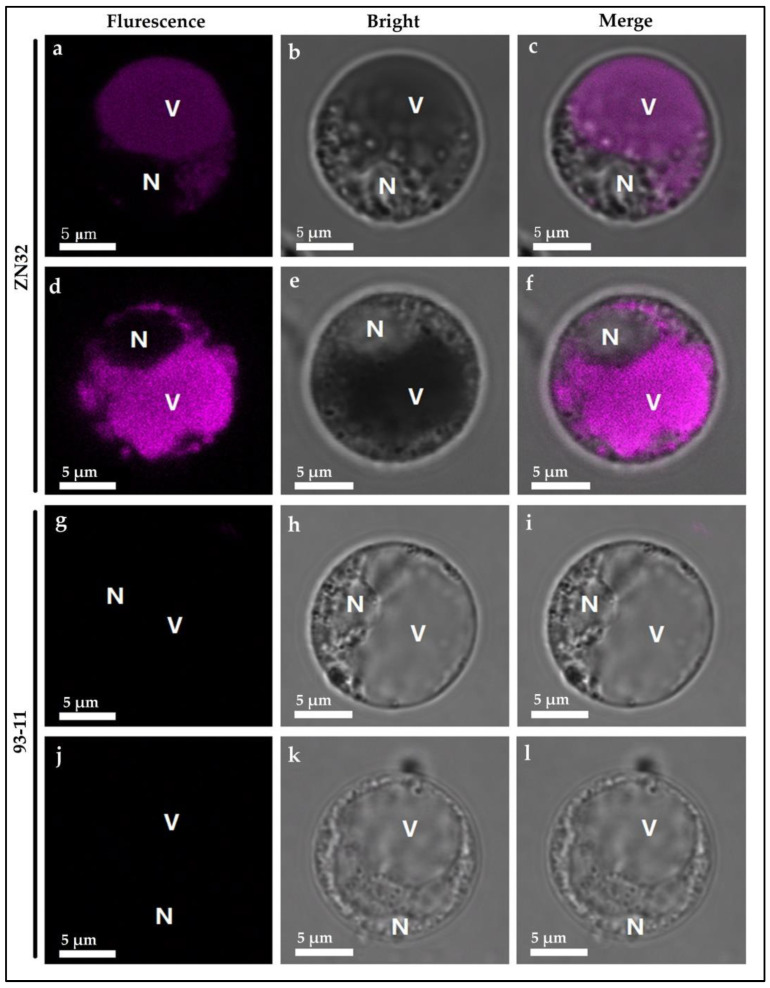
Phenotypic comparison of protoplasts extracted from ZN32 and 93-11 stigma under confocal microscopy. Note: (**a**–**f**), the protoplast of ZN32 showing fluorescence; (**g**–**l**), the protoplast of 93-11 showing no fluorescence; V indicates the position of vacuole; N indicates the position of nucleus in protoplast, bar = 5 μm.

To investigate the distribution of anthocyanin that can generate the differentially expressed cell morphology between ZN32 and 93-11. The stigma protoplast of ZN32 and 93-11 were extracted and evaluated by confocal microscopy according to previously reported research [50]. The vacuole in ZN32 showed significant reddish fluorescence coloration but no fluorescence signals were found in the nucleus, while no fluorescence signals were detected in the full cell in 93-11 (Figure 9). The results showed that anthocyanin, which is a kind of water-soluble pigment, was mainly distributed in the vacuole of the plant cell, which leads to the purple leaf margin phenotype in rice. In conclusion, this gene-controlling LMC is related to the synthesis of anthocyanin. It can be expressed specifically in some putative tissues, such as leaf margin, leaf sheath, stigma, apiculus, and so on (Figure 8 and Figure 9).

#### 3.10.3. Genetic Analysis and Mapping of the *qLMC6.1*

**Figure 10:**
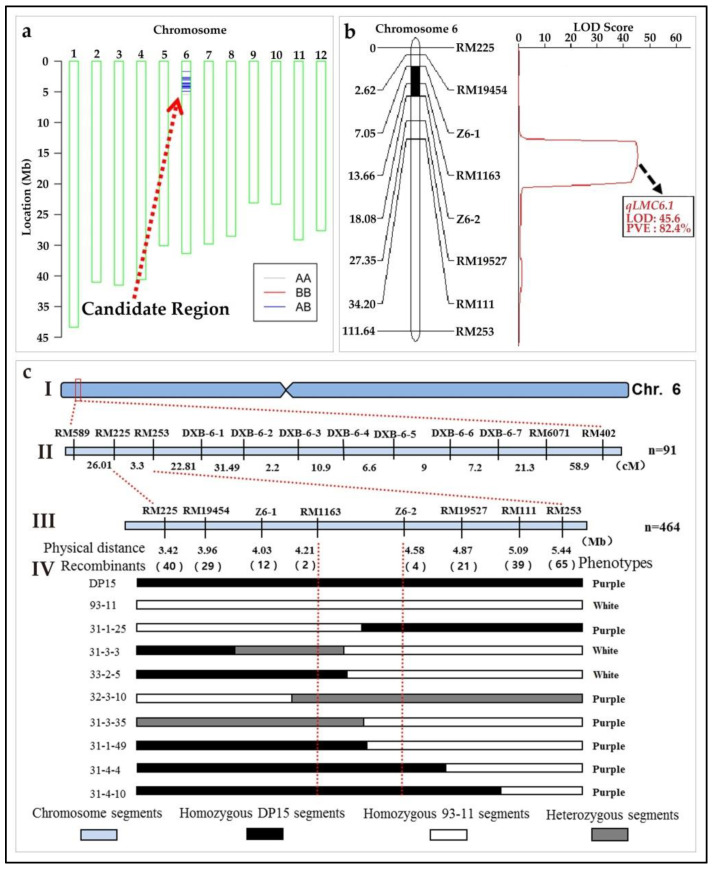
Genetic mapping of the *qLMC6.1* in rice. (**a**), the QTL detection figure of *qLMC6.1*; (**b**), the bulk segregation analysis for the *qLMC6.1*; (**c**), the recombinants identification and genetic mapping for the *qLMC6.1*; (I), simple model of Chromosome 6; (II), the distribution of primers used for primary mapping; (III), the distribution of primers used for fine mapping; (IV), the screening for recombinants for the mapping of *qLMC6.1*.

The chromosome segment substitution line ZN32, a homozygous CSSL with a purple leaf margin, is significantly different from that of the recipient parent 93-11. Through the genome background analysis of ZN32, it can be inferred that the gene locus *qLMC6.1* controlling the LMC is located in the interval of RM19381~DXB-6-4 on chromosome 6 (Table S15). The *qLMC6.1* was further mapped to the RM225~DXB-6-1 region by analyzing the substitution fragments of the adjacent substitution lines ZN31 and ZN33 that were consistent with its phenotypes (Figure 10). The secondary mapping population was constructed by backcrossing the substitution line ZN32 with the recipient parent 93-11. The results showed that the LMC of all the F_1_ generation was purple, while the LMC phenotypes in the F_2_ population were obviously separated. The phenotype data of the F_2_ population were recorded and analyzed by a Chi-square test (Table S13). The results showed that among the total 91 plants of the F_2_ population, 66 individuals showed purple leaf margin phenotype and 29 showed white leaf margin phenotype, which was consistent with the Mendel 3:1 segregation ratio (χ^2^ = 0.18 ≤ χ^2^_0.05,1_ = 3.84) (Table S13). Therefore, *qLMC6.1* may be encoded by a single locus. Through the 40K SNP microarray chip BSA method, *qLMC6.1* was further located in the overlapping fragments between the SNP molecular markers R0601663377 (1.66 Mb) and R0605432762TC (5.43 Mb) (Figure 10a, Tables S14 and S15). Through genetic linkage analysis of a secondary F_2_ population of 93-11/ZN32 by 12 SSR markers of Chromosome 6, *qLMC6.1* was initially mapped to the region of RM225~RM253 on the short arm of chromosome 6. The two linked markers, RM225 and RM253, were then used to screen recombinants of heterozygous type in the segregation populations of F_2_, the selected heterozygous recombinants ware subsequently self-crossing to obtain F_3_ segregation populations. Based on the results of high-throughput sequencing, six new polymorphic InDel markers between RM225 and RM253 were developed to conduct a fine mapping of *qLMC6.1*. Through the identification and analysis of the genotype and phenotype of 464 individuals of the F_3_ segregation population, it is confirmed that *qLMC6.1* is located in the 370 Kb region between marker RM1163 and Z6-2, with a LOD value of 45.6 and a PVE value of 82.4% (Figure 10b,c and Table S15).

## 4. Discussion

As one of the earliest domesticated cereal crops, rice feeds half of the world’s population. During the long-term domestication and natural selection, the presently cultivated rice showed remarkable morphological changes compared to common wild rice in evolution [64]. Through long-term artificial and natural selection in history, various genes of the cultivated rice have already been missed during the domestication courses, the relatively complete genome of wild rice ensures its wider phenotypic diversity in various traits. Although several novel QTLs were identified using CSSLs/SSSLs of cultivated rice [65,66,67], few wild rice CSSLs/SSSLs were developed for the mining of new genes [68,69]. Located in the subtropical zone, Guangxi is rich in wild rice resources [70,71]. Rice domestication through artificial and natural selection led to the reduction of several important agronomic traits that can be found in wild rice. Based on the extensive germplasm resources of Guangxi, a typical common wild rice accession DP15 with several important economic traits was identified and selected to develop a set of CSSLs (Figure S1). Our investigation revealed the significant phenotypic difference in various morphological traits observed between DP15 and the indica rice variety 93-11, including PH, awn length, leaf width, LMC, tiller number, tiller angle, spreading panicle, seed color, seed shattering, seed dormancy, GN, GL, GW, TGW, GLWR, and so on [72]. Through the WGRS, the genomic differences were highlighted by the bioinformatic analysis in this study, and 12,565 pairs of polymorphic InDel markers were designed to establish DP15-CSSLs and mining for novel genome QTLs. Both the extensive phenotypic and genetic variation make this DP15-CSSL a natural gene pool that can be utilized to identify new QTLs and generate rice cultivars with advantageous traits. As is known, CSSLs consist of a set of consecutive and overlapping donor chromosome segments in a recipient genetic background, which is an ideal genetic population for the mapping of QTLs [73,74]. In this study, 59 CSSLs from the common wild rice (*O. rufipogon* Griff.) accession DP15 under indica rice cultivar (*O. sativa* L. ssp. indica) variety 93-11 backgrounds were constructed through whole genome re-sequencing, multiple backcrosses, self-crossing, and MAS. The total genome substitution segment length of this DP15-CSSLs library was 344.34 Mb, and the average coverage rate of substitution segments in the chromosome was 91.72%. The genome coverage rate of the DP15-CSSLs can be increased with the expanded screening of CSSLs from the progeny of BC_4_F_2_, BC_5_F_2_, and BC_6_F_2_ progeny. In contrast to previous research on CSSLs, the DP15-CSSLs showed a higher coverage rate, which was mainly defined by the density and amounts of polymorphic molecular markers [75,76]. Moreover, our DP15-CSSLs library was constructed under the indica rice background, which will be complementary to the wild rice CSSLs research [77]. In recent years, several genes controlling the resistance to both biological stress and abiotic stress have been identified [78,79,80]. However, novel genes related to agronomy traits such as grain appearance, leaf color, and PH remain to be exploited. The molecular mechanisms of how these traits function are still largely unknown.

Besides the significant difference in phenotype, there are a large number of genomic variations between common wild rice and cultivated rice, which is of great convenience for the detection of QTLs. Parental materials that show phenotypic variation in the target traits due to variations in the genome are necessary for genetic QTL mapping [81]. With the rapid development in the technology of bioinformatic analysis and genome sequencing, extensive genomic SNPs and InDels can be well detected and applied to gene mapping and prediction. SSSLs/CSSLs with both higher genetic and phenotypic differences are effective tools for fine mapping, cloning, and analysis of novel QTLs [82,83]. CSSLs/SSSLs have previously promoted the identification of novel QTLs related to grain traits in Yuanjiang common wild rice species [84,85,86]. A set of SSSLs harboring the C563~C63 region encoding for long stigma was identified from Nipponbare/Kasalath-SSSLs and a secondary F_2_ population of SSSL14/Nipponbare was successfully used to fine-map the *qSTL3*, which identified *LOC_Os03g14850*, *LOC_Os03g14860*, and *LOC_Os03g14880* as the candidate genes controlling stigma length [87]. The study of wild rice traits, especially grain-related traits, is promising for further improvements in the yield and quality of cultivated rice [83]. Agronomy traits such as the GL, GW, and TGW are the major determinant of yield potential [88]. Through the phenotype screening of DP15-CSSLs, four-grain traits including GL, GW, TGW, and GLWR that are significantly different from 93-11 were selected for the detection, of novel QTLs. To decrease the influence of variation in the phenotypic values for QTLs detection, these four traits were recorded in different experiment fields over two years [89]. Thus, a total number of 20 QTLs were detected. Among them, seven QTLs controlling TGW were detected by the whole genome screening, of which *qTGW3.1* is near the gene *LPA1* (*LOC_Os03g13400*), which encodes a plant-specific transcriptional inhibitor associated with shorter grains and decreased TGW, the other QTLs are new QTLs without any previous report [82]. Based on the genotype and phenotypic values of these DP15-CSSLs on GL, five QTLs on GL were identified. The GL that often shows a positive correlation with GLWR is an important agronomy trait for grain appearance [90]. *The qGL3.1* detected in this research is near the long kernel controlling gene *OsGS3,* and *OsGS3* is the main factor controlling rice GL and TGW [91]. But the other four QTLs of GL are distributed in new regions according to the previously reported QTLs on GL [92]. Five QTLs related to GW were also detected by the whole genome screening, of which the *qGW6.1* detected in this DP15-CSSLs is near the previously cloned *DSG1* (*LOC_Os06g06090*) gene, *DSG1* belongs to the *OsMAPK6* family and results in dwarfing, shorter internodes, erect leaves, smaller anthers and grains, and a significant decrease in GL, GW, and TGW [93,94]. In addition, Three QTLs related to GLWR were detected through the whole genome screening of QTLs in this DP15-CSSLs. The *qGLWR7.1* detected in a region from RM6071 to RM400 was near the *OsGL7* gene, *GL7* encodes a LONGIFOLIA protein and results in an increased GLWR, larger and more dense starch granules [95]. However, the other two QTLs related to GLWR were novel QTLs according to previous studies [96]. The traits of grain morphology such as GL, and GW often show significant correlations with TGW in cereal crops. Interestingly, the QTLs *qGL1.1* and *qTGW1.1* were detected in a similar region that may be the same QTL. The *qGLWR1.1* and *qGW1.1* were detected in an overlapping region on chromosome 1, which may be affected by the significant correlations between GW and GLWR in cereal crops [97]. The *qGLWR7.1* and *qGW7.1* detected in chromosome 7 were linked to the same region near the RM429. Further experiments are being carried out for elucidation.

Besides the 20 QTLs related to grain traits, one dominant QTL *qPH1*.*1* controlling the PH on chromosome 1 and one novel dominant QTL *qLMC6.1* controlling LMC on chromosome 6 were detected. As the traits of long awn and shattering, higher PH and purple leaf margin are often typical characterizations of wild rice [98,99,100]. A homozygous long-culm DP15-CSSL line and a purple leaf margin DP15-CSSL line were selected to construct secondary genetic populations for the mapping of *qPH1*.*1* and *qLMC6.1*. Based on the genotype and phenotypic values of the secondary populations, the *qPH1*.*1* controlling higher PH was successfully validated and mapped to a region of 4.31 Mb and *qLMC6.1* associated with purple leave margin was located in a region of 370 kb. The *qPH1.1*-containing plants showed a long culm phenotype with a significantly increased length on the internodes of rice. The genetic basis of PH can mainly be affected by cell elongation and cell density in the unit area of stem cells [101]. To verify the underlying mechanism in the generation of the longer internode, the frozen and paraffin section of rice culm were conducted to detect the cell morphology in stems. The results of this research showed that *qPH1.1* can significantly promote cell proliferation in the stem to generate an increased PH. Our previous research revealed *sd1* gene controlling the PH mainly by the increase in cell size and cell layers was nearly located in the same region on chromosome 1 with *qPH1.1*. However, *qPH1.1* showed a higher PH than the *sd1* mutants, which implied that *qPH1.1* may be a novel allele controlling higher PH [102]. In terms of phenotype, the *qPH1.1* detected from DP15-CSSLs is novel compared to these previously mapped QTLs of PH in rice [103]. The *OsBRI1*, which showed a close linkage with the RFLP marker C1370, was also located near this region [104]. The mutant plants of *OsBRI1* showed a BR signal transduction inhibition, which caused the elongation limitation of specific internodes, the leaf angle decreased, the leaf blade was upright, and the leaf sheath was shorter than that of wild-type, the spike neck was longer than that of wild-type [105]. In contrast to *OsBRI1, qPH1.1* showed no significant difference in leaf sheath, leaf angle, and longitudinal cell elongation, which implied that the BR signal transduction pathway showed less effect on *qPH1.1* [106]. Our ongoing exploration of this *qPH1.1* will focus on the gene regulatory network by gene prediction and RNA-sequencing, which may disclose the potential mechanism [107]. The stem diameter of ZN6 is significantly larger than 93-11, which makes the ZN6 higher biomass and is resistant to lodging to a certain extent. The long-culm DP15-CSSL line ZN6 of higher biomass will provide an economic material for the animal husbandry industry such as frog farming, crab aquaculture, duck, and livestock breeding [38,39,40,108]. Many biomass-related QTLs of rice have already been detected by researchers worldwide [41,42]. Anthocyanin is attractive for its innate coloring, antioxidant capacity, and biological potential in food additives and functional foodstuffs [109,110]. The mining of *qLMC6.1* from wild rice will promote the exploration of the anthocyanin distribution in specific tissues. Up to now, several anthocyanin-related genes have already been cloned by researchers worldwide in plants [50,111]. Compared to the already mapped QTLs related to anthocyanin, the *qLMC6.1* detected in this research is located near the Os*C1* gene. The Os*C1* is critical for anthocyanin production in rice [111,112,113]. The *qLMC6.1* will be an important tool in selective breeding for pure varieties. To verify the underlying mechanism in the generation of the color, the stigma protoplast of ZN32 and 93-11 were isolated and evaluated by confocal microscopy to detect the distribution of anthocyanin in the plant cell. The results showed that anthocyanin which is a water-soluble pigment was mainly distributed in the vacuole of the plant cell may lead to the purple leaf margin phenotype in rice, which is consistent with previous studies [28,111]. The *qLMC6.1* controlling LMC is related to the synthesis of anthocyanin and tissue-specific expressed specifically in some putative tissues, such as leaf margin, leaf sheath, stigma, apiculus, and so on (Figure 8 and Figure S11). Our ongoing experiment on *qLMC6.1* will focus on gene prediction and cloning, the gene, and promoter of *qLMC6.1* are promising to explain the underlying the mechanism of anthocyanin regulatory network (Figure S15). SSSLs/CSSLs of wild rice, which possess great potential for further exploitation and utilization, are good breeding materials for future rice breeding and improvement [15,82]. In all, these 22 QTLs identified from Guangxi common wild rice can potentially promote theoretical knowledge and genetic applications to rice breeders worldwide.

## 5. Conclusions

In this research, a set of 59 CSSLs covering 91.72% of the wild rice DP15 genome with the indica rice cultivar 93-11 backgrounds were constructed. Significant differences in four grain-related traits, PH, and LMC phenotypes between the Guangxi wild rice DP15 and the 93-11 were identified for the QTL detection in this research. About 20 QTLs associated with grain-related traits, one PH-controlling QTL, and one LMC-regulating QTL were detected. Furthermore, 12,565 mapped InDels were identified and designed for polymorphic molecular markers by high-throughput genome re-sequencing between wild rice accession DP15 and indica rice cultivar 93-11, which are well-identified and designed for polymorphic molecular markers. The PH-controlling QTL *qPH1.1* and the LMC-regulating QTL *qLMC6.1* were fine-mapped by the construction of two secondary genetic populations, which are of great significance for breeding and gene cloning. Thus, the DP15-CSSLs are a promising tool for novel gene discovery and rice breeding. Our ongoing experiments aim to investigate the grain-size-related QTLs in wild rice and clone the novel QTLs mapped in this research.

## Data Availability

Raw data can be provided to researchers on request to corresponding or first author.

## Supplementary Materials

The following supporting information can be downloaded at: https://www.mdpi.com/article/10.3390/biom12121850/s1, Figure S1: Nursery figures of 2361 common wild rice germplasm; Figure S2: Roadmap used to establish genetic population of DP15-CSSLs; Figure S3: Morphology of several typical grain traits in DP15-CSSLs; Figure S4: Phenotype of the purple leaf margin CSSL line ZN32; Figure S5: The chromosomal distribution of reads detected on the genome of DP15; Figure S6: The regional distribution of mapped reads detected on the DP15 genome; Figure S7: Frequency distribution circos of InDels and SNPs of DP15 and 93-11; Figure S8: Density and distribution diagram of polymorphic InDels markers between DP15 and 93-11 based on the WGRS; Figure S9: Representative polyacrylamide denaturing gels electrophoretogram obtained from DP15 and 93-11 amplified with the InDels and SSR markers; Figure S10: Distribution of substituted segments length in 59 DP15-CSSLs; Figure S11: Distribution of twenty QTLs for four different grain traits on 12 chromosomes; Table S1: Polymorphic InDel makers designed between DP15 and 93-11 re-sequenced genome; Table S2: Polymorphic molecular markers used for the construction of DP15-CSSLs; Table S3: Distribution and density of polymorphic molecular markers for the construction of DP15-CSSLs; Table S4: The distribution of chromosome substitution segments in 59 DP15-CSSLs; Table S5: Average statistics for four grain traits of 93-11 and the 59 DP15-CSSLs populations observed over two years; Table S6: Whole-genome re-sequencing analysis of DP15 and 93-11; Table S7: Distribution and density of SNPs identified between DP15 and 93-11; Table S8: QTLs related to four grain traits of DP15 detected with the linkaged markers based on the DP15-CSSLs; Table S9: Comparison of internode length in culms between ZN6 and 93-11; Table S10: Comparison of internode diameter in culms between ZN6 and 93-11; Table S11: Genetic segregation analysis of its F_2_ population for the plant height controlling QTL *qPH1.1*; Table S12: Polymorphic molecular markers for the mapping of *qPH1.1*; Table S13: Genetic segregation analysis of the F_2_ population for the leaf margin controlling QTL *qLMC6.1*; Table S14: Polymorphic molecular markers for the mapping of *qLMC6.1*; Table S15: Genes prediction in associated region of *qLMC6.1* locus by genetic mapping.

## Abbreviations

CTAB	Hexadecyl Trimethyl Ammonium Bromide
PH	Plant Height
GW	Grain Width
GL	Grain Length
GN	Grain Number
TGW	Thousand-grain Weight
GLWR	Grain Length-width Ratio
LMC	Leaf Margin Color
AC	Apiculus Color
SC	Stigma Color
LSC	Leaf Sheath Color
SSR	Simple Sequence Repeats
SNP	Single Nucleotide Polymorphism
InDel	Insertion-Deletion
RFLP	Restriction Fragment Length Polymorphism
MAS	Marker-assisted Selection
PAGE	Polyacrylamide Gel Electrophoresis
PCR	Polymerase Chain Reaction
CSSLs	Chromosome Segment Substitution Lines
SSSLs	Single Segment Substitution Lines
NILs	Near Iso-genic Lines
DHs	Double Haploid Lines
LOD	Logarithm of The Odds
PVE	Phenotypic Variation Explained
QTL	Quantitative Trait Locus
BSA	Bulked Segregants Analysis Sequencing
Chr.	Chromosome
RNA-seq	RNA-Sequencing
WGRS	Whole Genome Re-sequencing
GWAS	Genome Wide Association Study
BSA	Bulked Segregants Analysis
GA	Gibberellin Acid
SEM	Scanning Electron Microscope
MAPK	Mitogen-activated protein kinase
bHLH	Basic Helix-loop-helix Transcription Factor
UTR	Untranslated Regions
CDS	Coding DNA sequence
ncRNA	Non-coding RNA
CAD	Cinnamyl Alcohol Dehydrogenase
Mt	Mitochondrion
Pt	Chloroplast
