# Supplementary material for: Detection of QTLs Regulating Six Agronomic Traits of Rice Based on Chromosome Segment Substitution Lines of Common Wild Rice (Oryza rufipogon Griff.) and Mapping of qPH1.1 and qLMC6.1
Source: Biomolecules. 2022 Dec 11;12(12):1850. doi: 10.3390/biom12121850 (PMC9775987; doi:10.3390/biom12121850)
Supplement: Supplementary file 1 [file biomolecules-12-01850-s001.zip › 1.Supplimental Figures..pdf]

Detection of QTLs Regulating Several Grain Traits based on Chromosome Segment Substitution Lines (CSSLs) of Guangxi Wild Rice (Oryza rufipogon Griff.) and Mapping of qPH1.1 and qLMC6.1

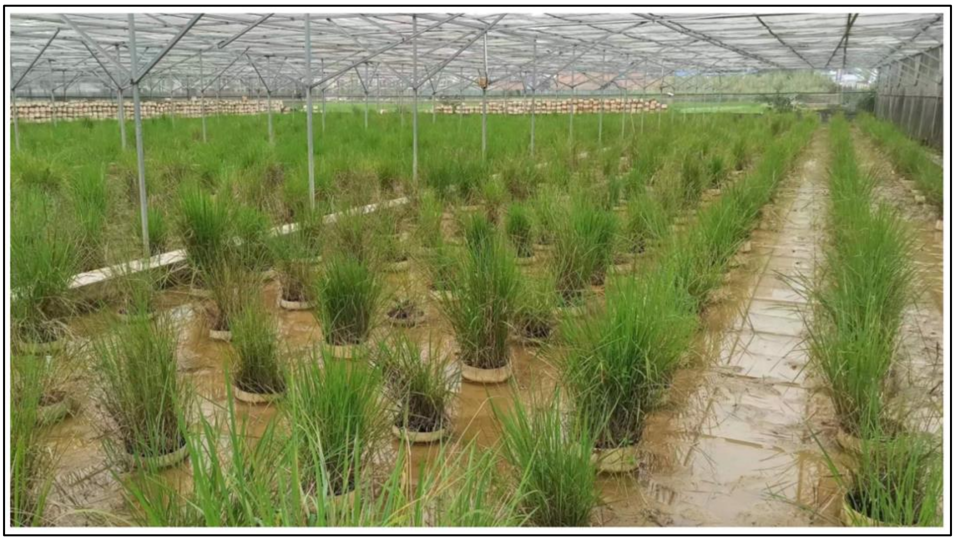

Figure S1. Nursery figures of 2361 common wild rice germplasm. Note: The germplasm resources garden belongs to State Key Laboratory for Conservation and Utilization of Subtropical Agro-Bioresources, Guangxi University, Nanning, China;

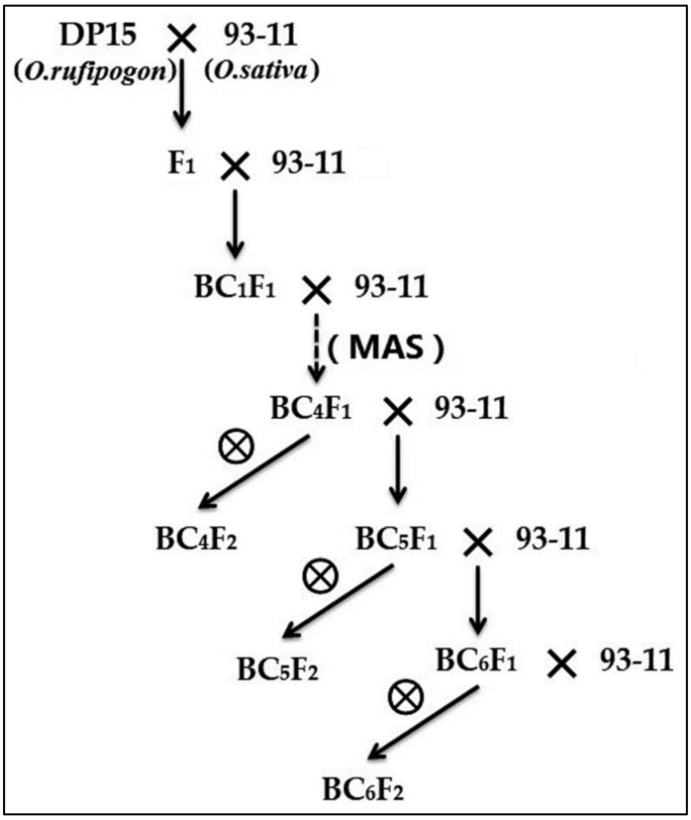

Figure S2. Roadmap used to establish genetic population of DP15-CSSLs. Note: MAS, molecular marker-assisted selection; ×, hybrid; ⊗, self-crossing.

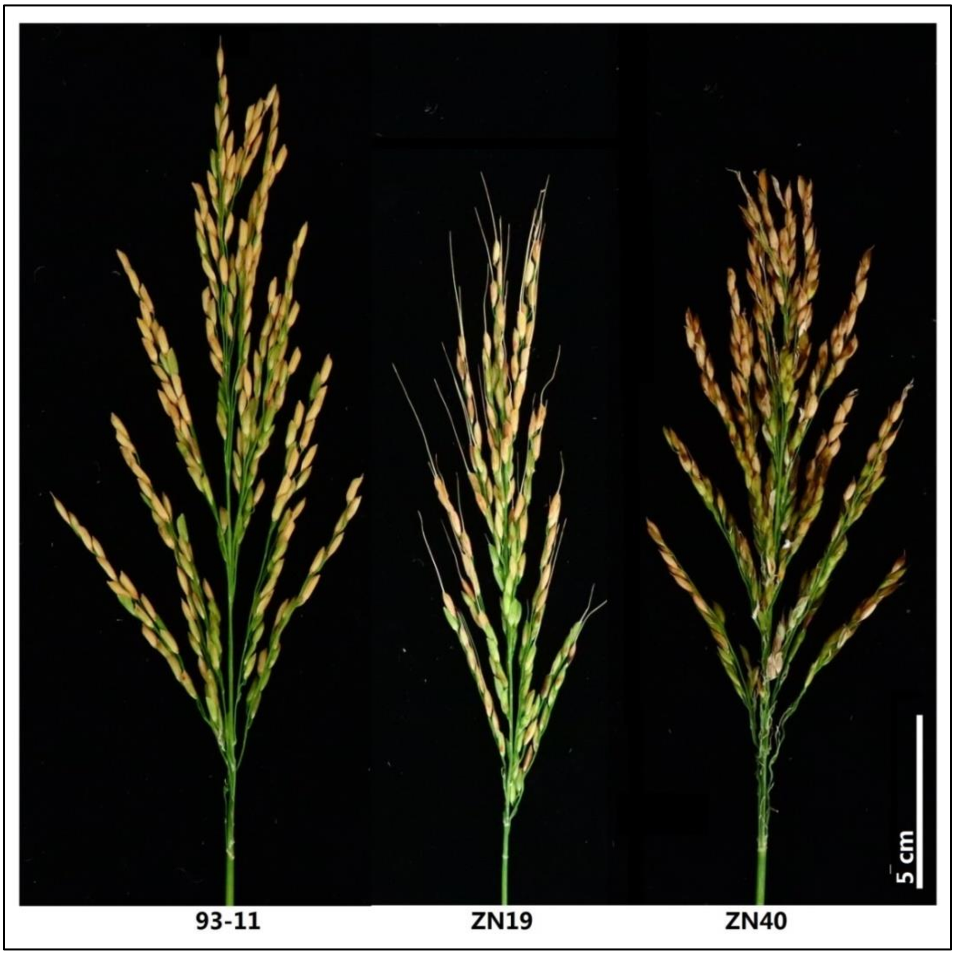

Figure S3. Morphology of several typical grain traits in DP15-CSSLs. Note: Bar = 5 cm.

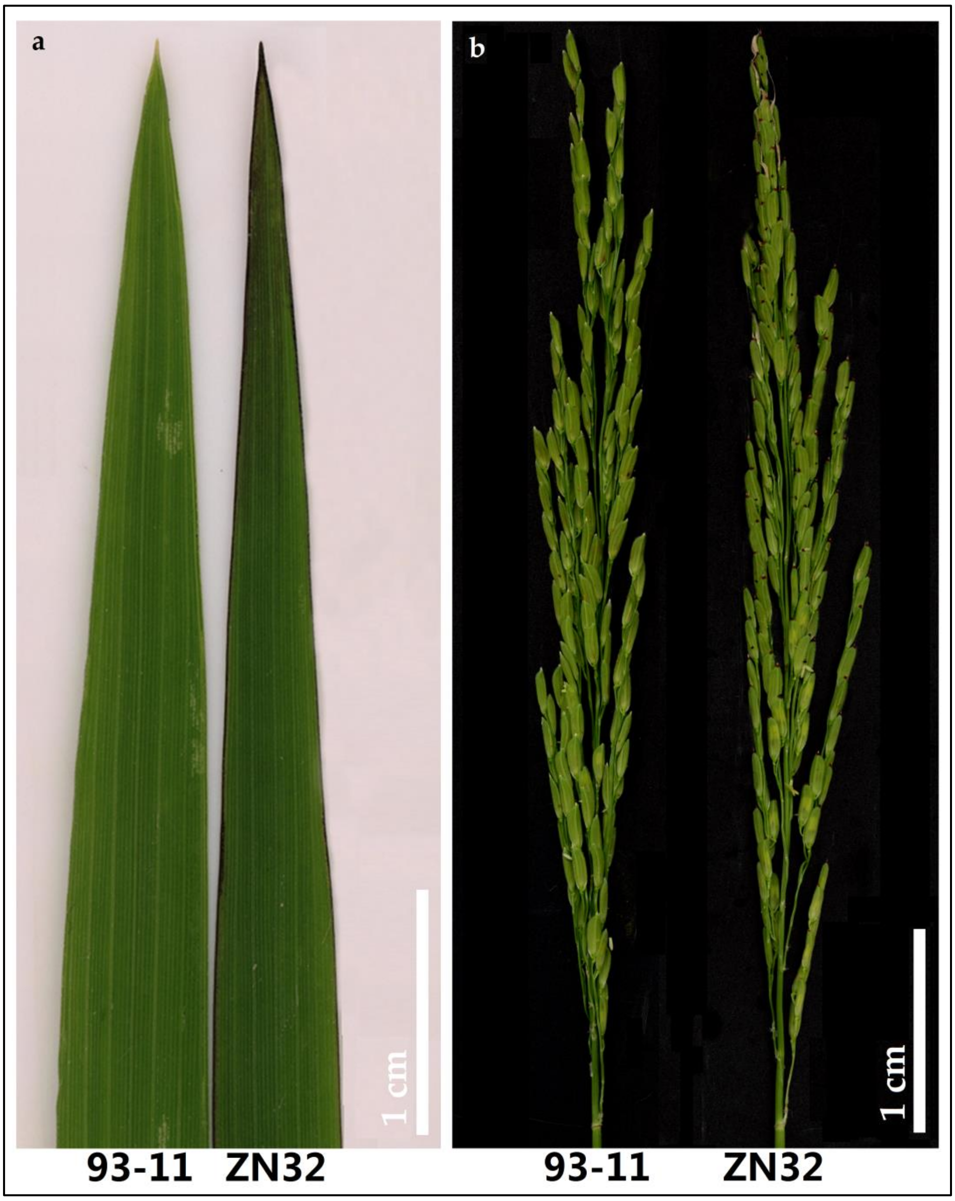

Figure S4. Phenotype of the purple leaf margin CSSL line ZN32. Note: (a), phenotype of leaf margin color of ZN32, bar = 1 cm; (b), phenotype of apiculus trait of in panicle of ZN32, bar = 5 cm.

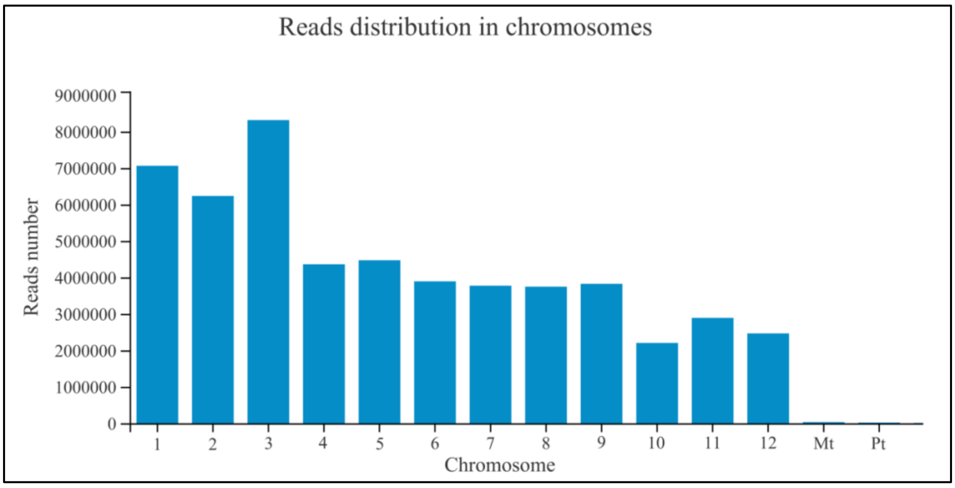

Figure S5. The chromosomal distribution of reads detected on the genome of DP15.Note: Mt, mitochondrion; Pt, chloroplast.

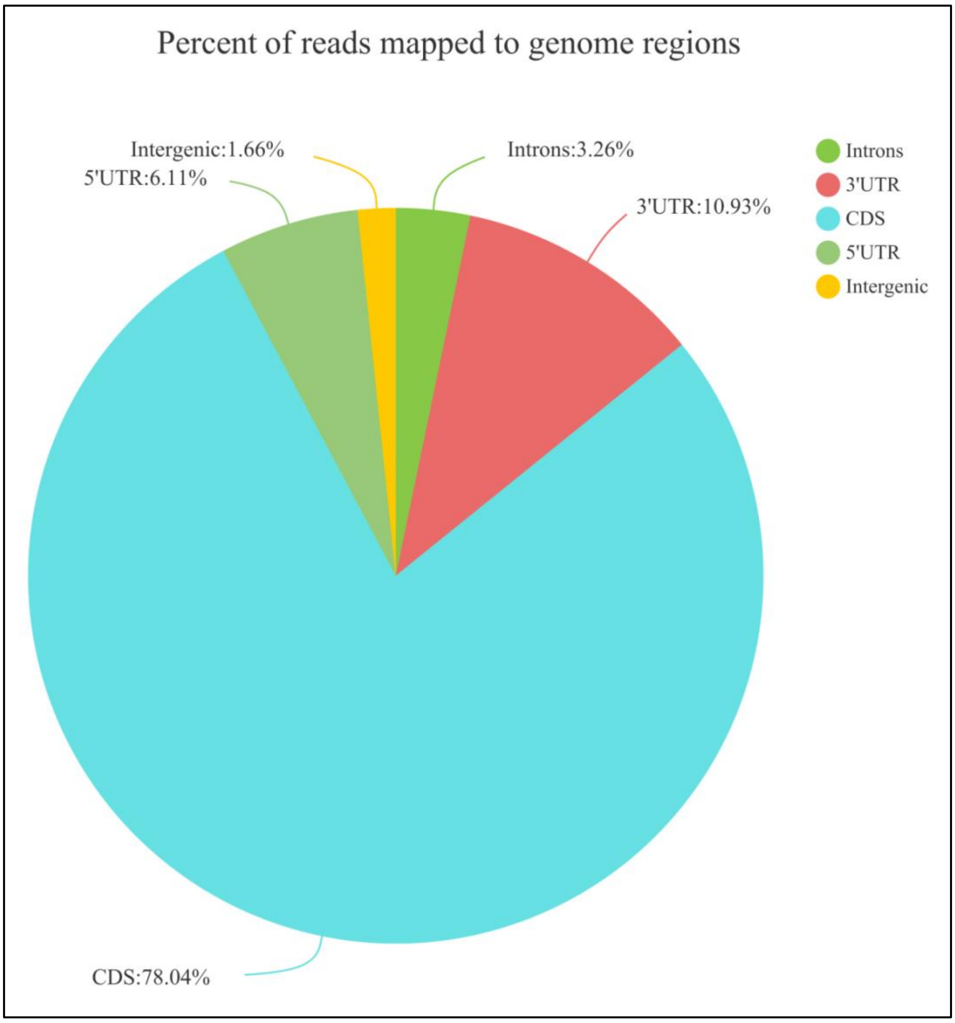

Figure S6. The regional distribution of mapped reads detected on the DP15 genome. Note: UTR, untranslated regions; CDS, coding DNA sequence.

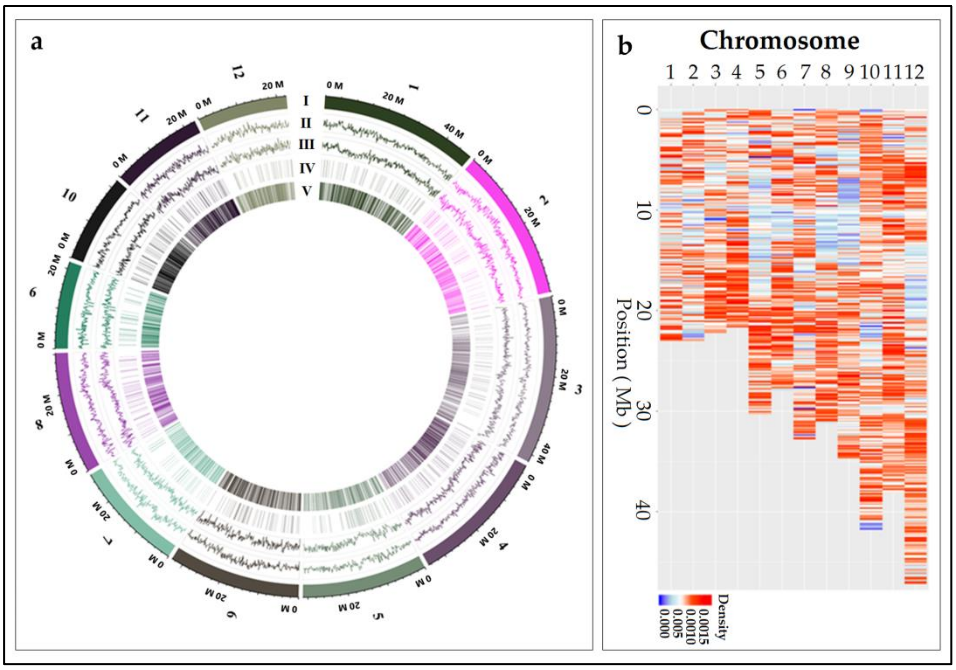

Figure S7. Frequency distribution circos of InDels and SNPs of DP15 and 93-11. (a), Comparative genomic analysis of SNPs and InDels between DP15 and 93-11 re-sequenced genome; (I), Cycle diagram with marked physic distance of 12 chromosome in rice genome; (II), the InDel density of 93-11; (III), the InDel density of DP15; (IV), the SNP density of 93-11; (V), the SNP density of DP15; (b), frequency distribution diagram of SNPs between DP15 and 93-11 on 12 chromosome.

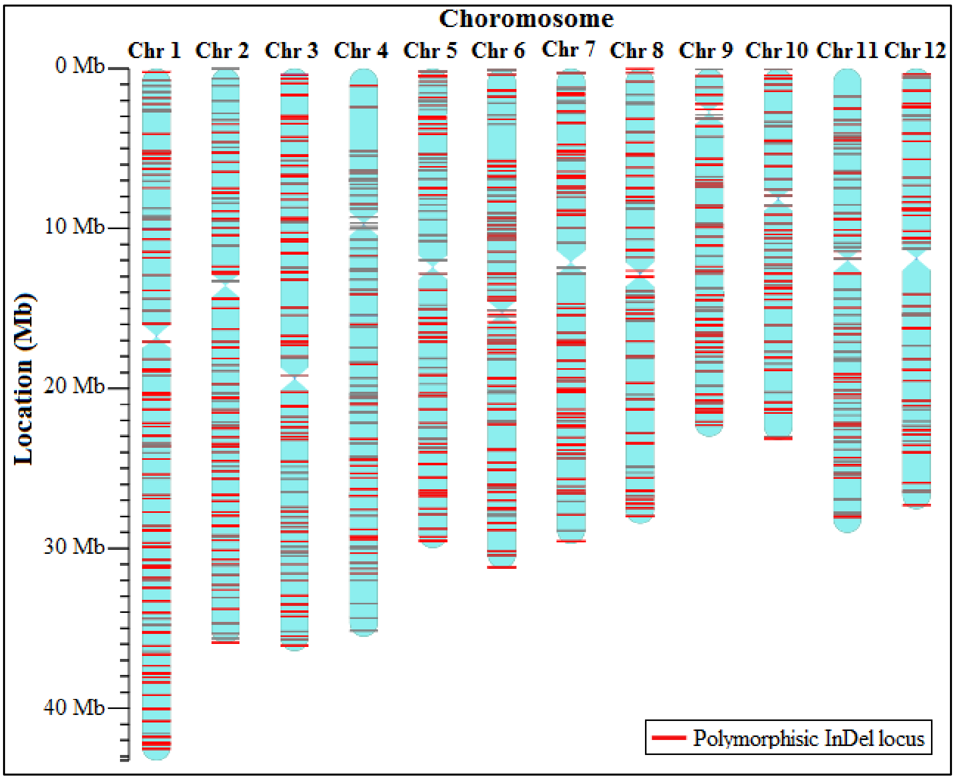

Figure S8. Density and distribution diagram of polymorphic InDels markers between DP15 and 93-11 based on the WGRS.

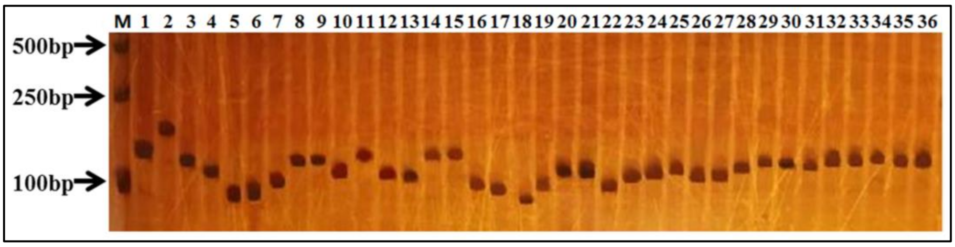

Figure S9. Representative polyacrylamide denaturing gels electrophoretogram obtained from DP15 and 93-11 amplified with the InDels and SSR markers. Note: The arrows indicate the laddered DNA markers; M, standard DNA marker lane; The electrophoresis lanes of 1, 3, 5, 7, 9, 11, 13, 15, 17, 19, 21, 23, 25, 27, 29, 31, 33 and 35 represents the DP30 bands amplified by molecular markers; The electrophoresis lanes of 2, 4, 6, 8, 10, 12, 14, 16, 18, 20, 22, 24, 26, 28, 30, 32, 34 and 36 represents the bands of 93-11 amplified by molecular markers.

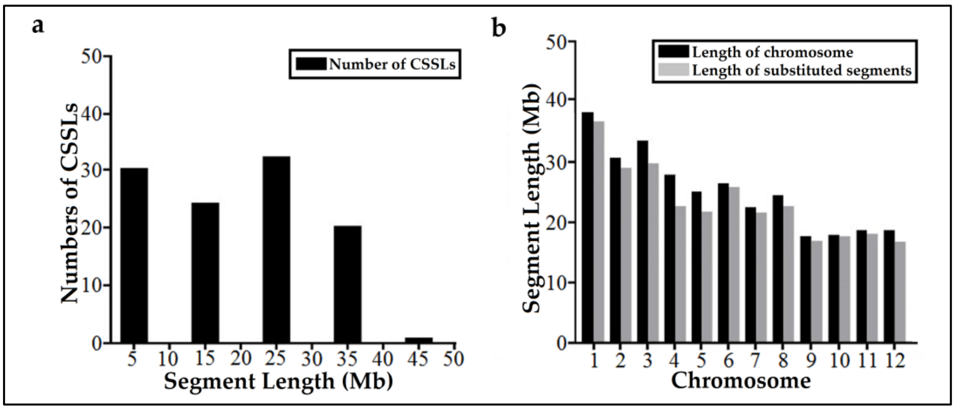

Figure S10. Distribution of substituted segments length in 59 DP15-CSSLs. Note: (a), distribution of the substituted segments length in DP15-CSSLs. (b), distribution of coverage substituted segments length in DP15-CSSLs on 12 chromosomes.

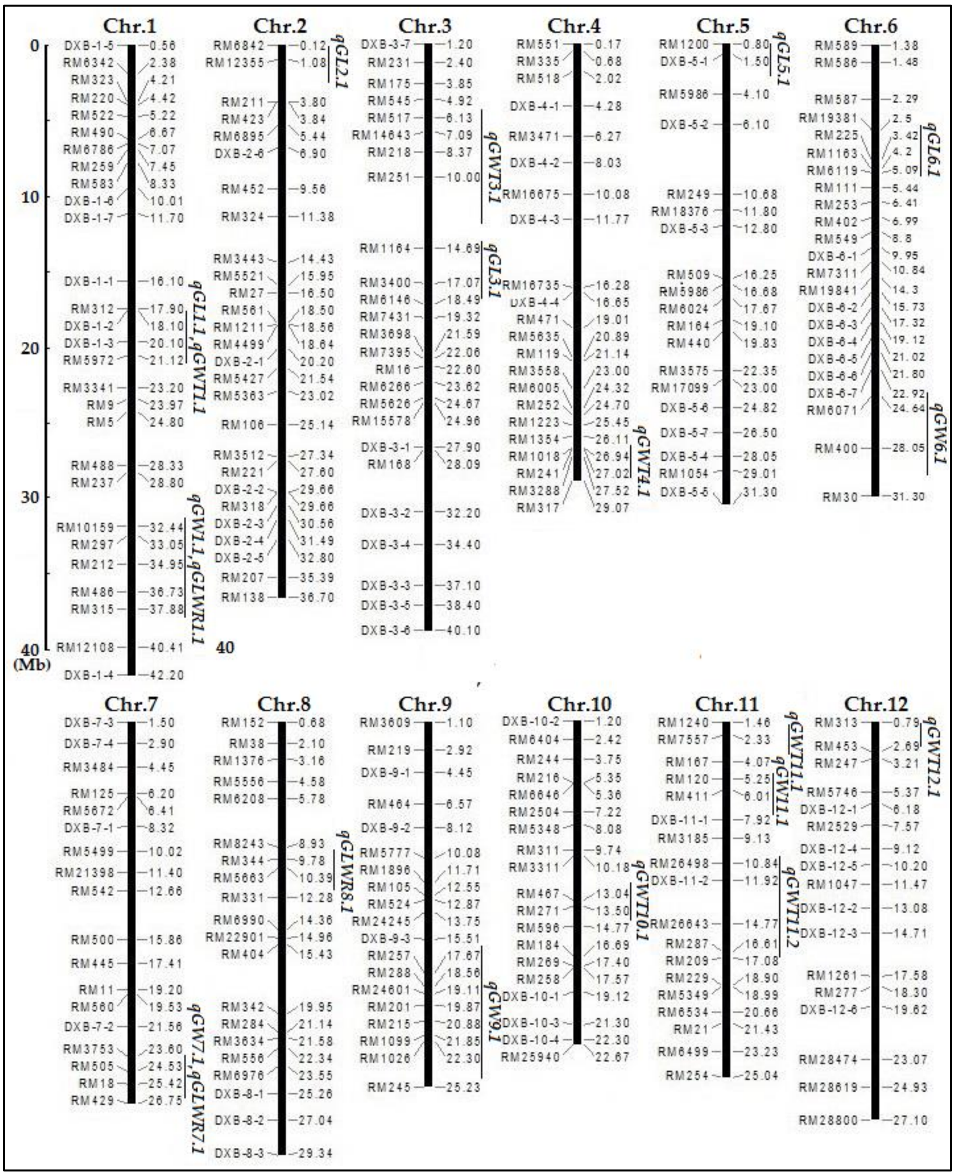

Figure S11. Distribution of twenty QTLs for four different grain traits on 12 chromosomes. Note: The molecular markers ware arranged on the left and the QTL locus was arranged on the right of each chromosome.
